# Threshold-Based Noise Detection and Reduction for Automatic Speech Recognition System in Human-Robot Interactions

**DOI:** 10.3390/s18072068

**Published:** 2018-06-28

**Authors:** Sheng-Chieh Lee, Jhing-Fa Wang, Miao-Hia Chen

**Affiliations:** Department of Electrical Engineering, National Cheng Kung University, No. 1, Dasyue Road, Tainan City 701, Taiwan; wangjf@mail.ncku.edu.tw (J.-F.W.); tanchi2@yahoo.com.tw (M.-H.C.)

**Keywords:** automatic speech recognition, noise detection, noise reduction, human-robot interaction

## Abstract

This work develops a speech recognition system that uses two procedures of proposed noise detection and combined noise reduction. The system can be used in applications that require interactive robots to recognize the contents of speech that includes ambient noise. The system comprises two stages, which are the threshold-based noise detection and the noise reduction procedure. In the first stage, the proposed system automatically determines when to enhance the quality of speech based on the signal-to-noise ratio (SNR) values of the collected speech at all times. In the second stage, independent component analysis (ICA) and subspace speech enhancement (SSE) are employed for noise reduction. Experimental results reveal that the SNR values of the enhanced speech exceed those of the received noisy speech by approximately 20 dB to 25 dB. The noise reduction procedure improves the speech recognition rates by around 15% to 25%. The experimental results indicate that the proposed system can reduce the effect of noise in numerous noisy environments and improve the quality of speech for recognition purposes.

## 1. Introduction

Automatic speech recognition (ASR) provides a user-friendly means of efficiently convey commands or requests to devices of human-machine interface (HMI). These devices can automatically analyze the received data and behave toward humans in ways that are consistent with the recognition results. A substantial literature exists on data classification and incomplete data analysis to reduce imputation error [1,2]. In recent years, research into ASR has considered many scenarios and applications. Much of the literature involves ASR for intelligent human-robot interaction [3,4,5,6,7]. When an ASR system is used in a real environment, especially a noisy one, the environmental noise considerably influences the quality of speech. The ambient noise can affect the signal components of speech and worsen the representation of recognition result. To solve the problem of noise, many methods of mitigating the effect of noise on ASR development, have been developed [8,9,10,11,12,13,14,15,16,17,18].

Choi et al. [8] presented a speech enhancement and recognition method for service robots. Their proposed adaptive beamformer structure includes a circular microphone array that comprised eight microphones. Jung et al. [9] presented a speech acquisition and recognition system, which can be utilized in home-agent robots. They used a generalized sidelobe canceller-based (GSC-based) algorithm with a microphone array to compensate the effect of room reverberation. Betkowska et al. [10] studied a factorial hidden Markov model (FHMM), which can be combined with HMMs of clean speech and noise, to increase speech recognition accuracy in noisy environments. Gomez et al. [11] proposed a spectral subtraction-based method to eliminate room reverberation for human-machine interaction. Ohashi et al. [12] demonstrated the use of a spatial subtraction array for noise reduction and employed the noise superimposition to realize a hands-free device of speech recognition. Hong et al. [13] executed a multi-channel GSC-based speech enhancement algorithm and designed a masking-based Wiener filter to reduce the residual noise. A noisy environment-aware speech recognition system has been used in human-robot interaction [14]. The system can determine whether the speech should be enhanced from the initial SNR value of the speech in noisy environments. Mohammadiha et al. [15] presented a speech enhancement method with a Bayesian formulation of nonnegative matrix factorization (BNMF). They adopted a scheme to train the noise BNMF model; the training BNMF model can be used as an unsupervised speech enhancement system. A review of the aforementioned methods [8,9,10,11,12,13,14,15] indicates that they mainly improve speech recognition by reducing the effects of ambient noise or room reverberation. However, noise signals can have numerous properties in real-world situations. When noise signals are unknown, the noise reduction method, which is based on known noise information [10,12,15], cannot be used. In the previous work, [14], the system estimated only the initial SNR value in the noisy environment. The method may be affected when the noise is varied.

To make the ASR system more robust in noisy environments, the methodology of artificial neural networks (ANN), especially deep neural networks (DNN), has been widely utilized in speech enhancement for ASR in recent years [16,17,18]. The goal of DNN is to implement complex nonlinear numeric functions, which are used to directly map log-likelihood spectral features of noisy speech into corresponding clean speech. In DNN model training, several research studies develop the strategy of multi-style training on mixed speech and noise data. Although DNN-based methods can achieve high accuracy improvement in ASR, the DNN models require more training data to synthesize, the amount of training data even more than HMM-based systems.

Integrating the above-mentioned ASR methods, two aspects are considered in this work; the first one is the method of noise reduction, and the second one is the manner of training data. In noise reduction method, this work attempts to develop a blind source separation-based (BSS-based) method to remove the ambient noise. Since ambient noise is unknown and varied in numerous environments, the noise reduction method which does not require noise information is adequate to separate the noise from the noisy speech. To improve the quality of speech for recognition, another speech enhancement method is combined with BSS-based method. Closely investigating different noise situations, noise signals may not be obvious within several time intervals when noise signals are intermittent. In this case, noise reduction cannot be used because the over-filtering speech may cause speech distortion and reduce the speech recognition rate. To prevent the circumstance of over-filtering speech, a preprocessing scheme called threshold-based noise detection is proposed in this work. The proposed scheme can automatically determine that when noise should be eliminated according to the magnitude of the noise. With respect to training data, an HMM-based training system is used in this work due to the amount of training data and training time. This work implements the HMM-based training system by using hidden Markov model toolkit (HTK) [19]. The proposed system utilizes the HTK as a speech recognizer in speech recognition. Compared with the DNN-based system, the HMM-based system using HTK can usefully reduce the amount of training data because the recognizer is trained with only the clean speech.

According to the two aspects, this work presents an HMM-based speech recognition system for human-robot interactions in noisy environments. The system can be divided into two procedures, the first one is proposed threshold-based noise detection, and the second one is combined noise reduction. The system has following four properties: Training data requires only clean speech data, a proposed preprocessing scheme to prevent the over-filtering speech, noise reduction without predicted noise information, and valid effect on reducing ambient noise and improving speech quality. This work provides another feasible method for ASR system in noisy environments.

This paper is organized as follows. Section 2 provides an overview of the proposed recognition system. Section 3 describes in detail the proposed threshold-based noise detection and combined noise reduction procedure. Section 4 considers the experimental results. Section 5 briefly draws conclusions.

## 2. System Overview

**Figure 1:**
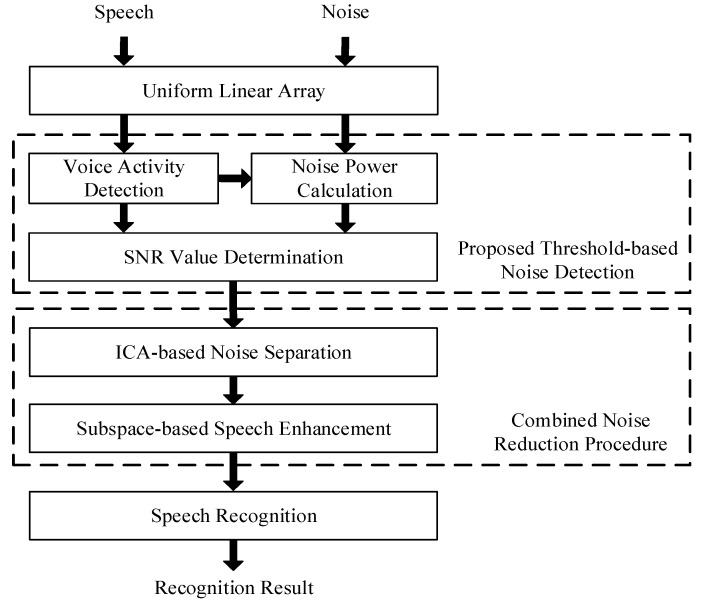
Overview of proposed system.

Figure 1 shows an overview of the proposed system. First to obtain information about the initial noise power, a linear array of two microphones is used to receive the initial noise signal in a noisy environment. When the noise signal is recorded, its power can be estimated by using a noise power calculation. From a noisy speech recording, the noisy speech signal can be identified as a speech signal or a non-speech signal (noise signal), based on the results of voice activity detection (VAD). If the collected signal is identified as a noise signal, then the noise power data can be updated from the power estimation of the current noise signal. Subsequently, the signal is used to determine the SNR value. The noisy speech signal can be input to the noise reduction procedure or speech recognition procedure as determined by comparative SNR determination.

Following the noise reduction procedure, an ICA-based method [20] is adopted to separate the noise signal from the noisy speech signal. However, the signal that is separated in the ICA processing retains the residual noise signal. To reduce the effect of the residual noise on the noisy speech signal and to reduce the speech distortion, a method of subspace-based speech enhancement [21] is applied after the ICA processing.

In speech recognition, an HTK-based speech recognizer, which is trained with clean speech data, is used in speech recognition. The recognizer analyzes and takes the approximate content of speech, which is the recognition result of the recognition system.

## 3. Proposed Methods

### 3.1. Proposed Threshold-Based Noise Detection

**Figure 2:**
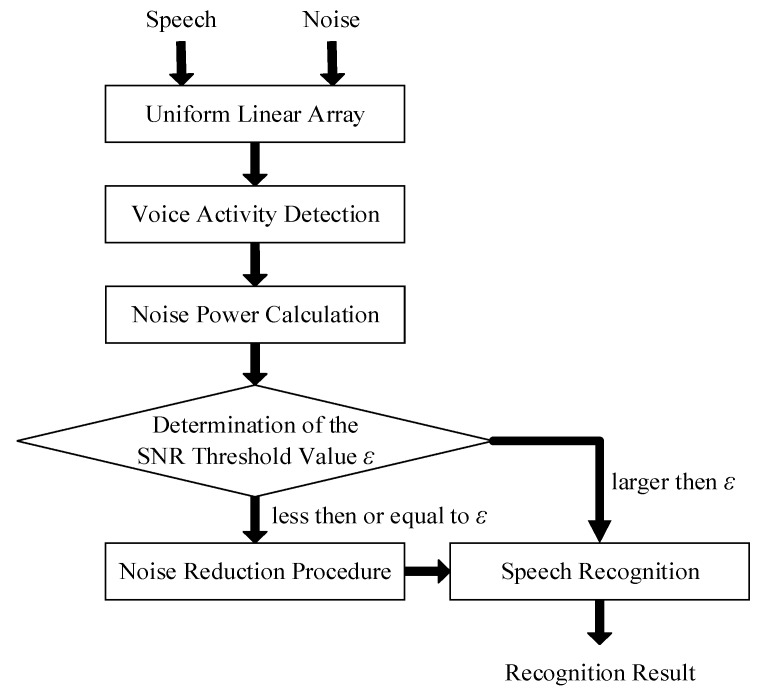
Procedure of proposed threshold-based noise detection.

Figure 2 displays the procedure of proposed threshold-based noise detection. A linear array is employed to collect the speech signal in a noisy environment. In the time domain, the observed signals *x*_1_(*t*) and *x*_2_(*t*) can be modeled as matrices and vectors in (1) and (2), where *y*(*t*) and *n*(*t*) denote the clean speech signal and the noise signal, respectively. Since the observed signal *x*_1_(*t*) is similar to *x*_2_(*t*), signal *x*_1_(*t*) is taken as the principal signal in the subsequent VAD, noise power calculation, and SNR determination.

(1) X=Y+N,

(2) $$\left[\begin{array}{c} x_{1}(t)\\ x_{2}(t) \end{array}\right]=\left[\begin{array}{c} y_{1}(t)\\ y_{2}(t) \end{array}\right]+\left[\begin{array}{c} n_{1}(t)\\ n_{2}(t) \end{array}\right]$$

The objective of VAD is to locate the speech signal component of the received signal. Two features, which are called short-time energy and zero-crossing rate (ZCR), are executed in VAD. The short-time energy is formulated as (3), where *w*(*n*) is the selected window function, and *L* is the length of the window. In the proposed system, the default window function is a Hamming window, which is defined in (4). The signal that has high amplitude can be found and treated as a speech signal. To detect the speech signal accurately, another feature, ZCR, is used in VAD.

(3) $$E_{x}=\sum_{n=0}^{L-1}{\left[x_{1}(n)w(n)\right]}^{2},$$

(4) $$w(n)=0.54-0.46\cos\left(\frac{2\pi n}{L-1}\right),\;0\leq n\leq L-1$$

Equation (5) represents ZCR; *z*(*t*) equals one if the amplitude of the observed signal *x*_1_(*t*) is positive and zero otherwise. ZCR can be used to discover the voiced signal, which has a lower ZCR than an unvoiced signal or noise. In VAD, the non-speech signal, which has a lower short-time energy and higher ZCR, can be regarded as a noise signal and be used in the noise power calculation.

(5) $$ZCR_{x}=\frac{1}{L-1}\sum_{t=1}^{L-1}\left|z(t)-z(t-1)\right|$$

The purpose of the noise power calculation is to estimate and update the mean power of the noise signal, which is detected in VAD. Equation (6) presents the relationship between the average noise power *P_n_* and the noise signal *n*(*t*). The mean noise power can be used in the following determination of the SNR threshold value.

(6) $$P_{n}=\frac{1}{L}\sum_{t=1}^{L-1}{\left|n(t)\right|}^{2}$$

In the determination of the SNR threshold value, the SNR value of the collected speech can be estimated. Equation (7) represents SNR, where *P_y_* and *P_x_* are the mean power of clean speech and noisy speech. The proposed system sets a threshold value *ε*, which is compared with the SNR value. When the SNR value is less than or equal to *ε*, the received speech should be enhanced because the power of noise signal is obvious. If the SNR exceeds *ε*, it means the effect of the noise on the collected speech is not obvious, and the collected speech can be directly passed to the speech recognition process.

(7) $$\begin{array}{lll} \varepsilon_{SNR} & = & 10\log_{10}\frac{P_{y}}{P_{n}}\\ & = & 10\log_{10}\frac{P_{x}-P_{n}}{P_{n}} \end{array}$$

### 3.2. Combined Noise Reduction Procedure

The combined noise reduction procedure comprises ICA and SSE. The observed signals in (1) and (2) can be expressed using an unknown mixing matrix **A**, which is in (8). The speech signal *y*(*t*) and the noise signal *n*(*t*) are regarded as the original source signals.

(8) $$\begin{array}{lll} X & = & \left[\begin{array}{c} x_{1}(t)\\ x_{2}(t) \end{array}\right]\\ & = & \left[\begin{array}{cc} a_{11} & a_{12}\\ a_{21} & a_{22} \end{array}\right]\left[\begin{array}{c} y(t)\\ n(t) \end{array}\right] \end{array}$$

Consistent with (8), to obtain the individual source signal from the received signals *x*_1_(*t*) and *x*_2_(*t*), a de-mixing matrix is estimated. Equation (9) represents the de-mixing matrix, where *s*_1_(*t*) and *s*_2_(*t*) are separated signals, and matrix **W** is the de-mixing matrix. The separated signals are similar to the original source signals.

(9) $$\begin{array}{lll} S & = & \left[\begin{array}{c} s_{1}(t)\\ s_{2}(t) \end{array}\right]\\ & = & \left[\begin{array}{cc} w_{11} & w_{12}\\ w_{21} & w_{22} \end{array}\right]\left[\begin{array}{c} x_{1}(t)\\ x_{2}(t) \end{array}\right]\\ & = & WX \end{array}$$

To calculate the de-mixing matrix, ICA exploits high-order statistics and information theory to measure the non-Gaussian characteristic of the property. The analysis of the non-Gaussian characteristic can be used to obtain the de-mixing matrix. In the ICA process, both source signals must be mutually independent. To solve the situation of mutually independent, two methods called signal centering and signal whitening are utilized in ICA. These methods ensure that the source signals can become uncorrelated. Signal centering is performed using (10), where **X** is the received signal, and *E*[**X**] is the mean of the received signal.

(10) $$\bar{X}=X-E[X]$$

The purpose of signal whitening is to evaluate a “whitening matrix”. The signal data that is described using (10) can become uncorrelated when multiplied by the whitening matrix. Equations (11) and (12) represent the whitening process in which **H** is a whitening matrix; $E[\hat{X}{\hat{X}}^{T}]$ is the covariance matrix of the signal, and **I** is the identity matrix.

(11) $$\hat{X}=H\bar{X},$$

(12) $$E[\hat{X}{\hat{X}}^{T}]=I$$

ICA adopts negentropy maximization to analyze the non-Gaussianity property of the signal. Equation (13) describes the formula for negentropy calculation, where the Gaussian distribution of signal ${\hat{Y}}_{\mathrm{Gauss}}$ has the identical covariance matrix as the estimated signal $\hat{Y}$, and the entropy is H(⋅).

(13) $$J(\hat{Y})=H({\hat{Y}}_{\mathrm{Gauss}})-H(\hat{Y})$$

To accelerate the ICA, the proposed system uses an algorithm that is called Fast ICA [22]. The calculation of negentropy can be approximately written as (14), where *α* and *β* are constants; $\hat{\gamma }$ is a zero-mean Gaussian variable with a standard deviation of unity, and $G_{1}(\cdot )$ and $G_{2}(\cdot )$ are contrast functions. Several functions, given by (15)–(17), can be taken as the contrast function in the negentropy calculation. The coefficient that is given by (15) is a constant with a value of between one and two.

(14) $$J(\hat{Y})\approx \alpha {\left(E[G_{1}(\hat{Y})]\right)}^{2}+\beta {\left(E[G_{2}(\hat{Y})]-E[G_{2}(\hat{\gamma })]\right)}^{2},$$

(15) $$G_{1}(x)=\frac{1}{a}\log(\cosh(ax)),$$

(16) $$G_{2}(x)=-e^{-\frac{x^{2}}{2}},$$

(17) $$G_{3}(x)=x^{4}$$

According to (14), when a single contrast function is used, the calculated negentropy can be proportional to a perfect square form, which is composed of the contrast function of the estimated signal $\hat{Y}$ and the zero-mean Gaussian variable $\hat{\gamma }$. Equation (14) can be rewritten as (18) and (19).

(18) $$J(\hat{Y})\propto {\left(E[G(\hat{Y})]-E[G(\hat{\gamma })]\right)}^{2},$$

(19) $$J(\hat{Y})\propto {\left(E[G(W^{T}\hat{X})]-E[G(\hat{\gamma })]\right)}^{2}$$

Equation (19) indicates that the maximum negentropy can be obtained by determining the maximum $E[G(W^{T}\hat{X})]$. The de-mixing matrix can be derived from the Newton iteration. The de-mixing matrix is represented as (20), where g(⋅) and $g^{\prime }(\cdot )$ are the derivatives of contrast functions G(⋅) and g(⋅).

(20) $$W\leftarrow E[\hat{X}g(W^{T}\hat{X})]-E[g^{\prime }(W^{T}\hat{X})]W$$

After the ICA processing, the two separated signals can be judged as the speech signal and the noise signal according to ZCR. Although ICA can separate noise from received signals, the separated signals retain the residual noise component. To remove the residual noise, the proposed system incorporates another technique, SSE, to design a filter and eliminate the effect of residual noise on the separated signals. The filter coefficients can be evaluated by subtracting the original speech signal from the filtered speech signal. Equation (21) describes this signal subtraction, where **F** denotes the SSE filter, and **I** is the identity matrix. Finally, the signal subtraction can be rewritten in terms of two parameters ***δ****_y_* and ***δ****_n_*. The former parameter specifies the speech distortion from the filter, and the latter specifies the residual noise after the filter process.

(21) $$\begin{array}{lll} \delta & = & FX-Y\\ & = & F\left(Y+N\right)-Y\\ & = & (F-I)Y+FN\\ & = & \delta_{y}+\delta_{n} \end{array}$$

To optimize the filter process based on (21), the variances of speech distortion and residual noise, which are given by (22) and (23), are used to evaluate the filter coefficients.

(22) $${\bar{\delta }}_{y}=E[\delta_{y}{}^{T}\delta_{y}],$$

(23) $${\bar{\delta }}_{n}=E[\delta_{n}{}^{T}\delta_{n}]$$

In the filter coefficients evaluation, the recognition rate can be reduced when the speech distortion is obvious. Therefore, the speech distortion should be minimized as much as possible. The residual noise also can influence the recognition result. To prevent this situation, the residual noise should be suppressed. With respect to the speech distortion and the residual noise, the two aforementioned requests can be defined as follows:

(24) $$\arg\underset{F}{\min}\;{\bar{\delta }}_{y},$$

subject to

(25) $${\bar{\delta }}_{n}\leq \gamma \sigma_{n},$$

where $\sigma_{n}$ is the variance of the noise signal, and *γ* is the adjustable parameter, whose value is between zero and one.

Consistent with (24) and (25), the optimal filter is obtained using the Lagrange multiplier method. The optimal filter is represented as (26), where **R_YY_** and **R_NN_** are the covariance matrices of the speech signal and the noise signal, respectively, and *μ* is a Lagrange multiplier.

(26) $$F=R_{YY}{\left(R_{YY}+\mu R_{NN}\right)}^{-1}$$

The covariance matrix **R_YY_** described in (26) can be given by (27) using eigenvalue decomposition (EVD), where **Q** is a square matrix whose *i*th column is the eigenvector **q***_i_*, and **Λ_YY_** is a diagonal matrix.

(27) $$R_{YY}=Q\Lambda_{YY}Q^{-1},$$

substituting (27) into (26) yields,

(28) $$F=Q\Lambda_{YY}{\left(\Lambda_{YY}+\mu Q^{-1}R_{NN}Q\right)}^{-1}Q^{-1},$$

expressing **R_NN_** in EVD form enables (28) to be rewritten as (29).

(29) $$F=Q\Lambda_{YY}{\left(\Lambda_{YY}+\mu \Lambda_{NN}\right)}^{-1}Q^{-1}$$

### 3.3. Speech Recognition Process

The proposed system utilizes the HTK as a speech recognizer in speech recognition. About the selection of speech corpus, the system takes corpora of Mandarin speech data across Taiwan (MAT-400) to train acoustic models, numerous acoustic models have been trained in the HTK recognizer. For feature extraction of speeches, the HTK uses Mel-frequency cepstral coefficients (MFCCs) as the speech features in speech recognition. In recognition process, the HTK-based speech recognizer analyzes the speech features and selects the most appropriate content of speech as the recognition result.

## 4. Experimental Results

### 4.1. Experimental Setup

**Figure 3:**
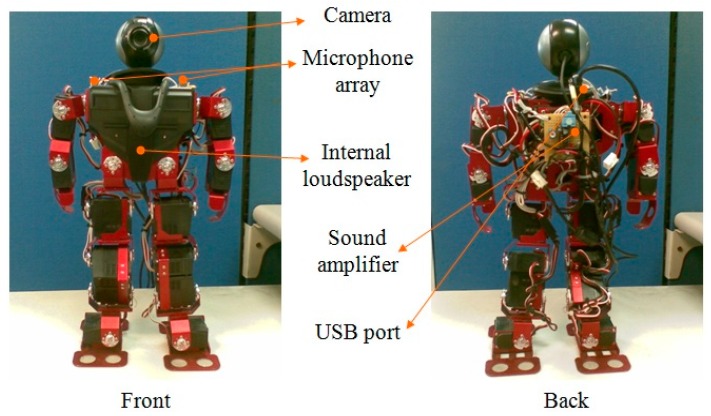
Humanoid robot 16-DOF Robotinno^TM^.

To realize the proposed system, a humanoid robot, called 16-DOF Robotinno^TM^, is used herein. Figure 3 shows the illustration of the robot. With respect to the linear array, two omni-directional microphones are placed with a spacing of 0.1 m on the shoulder of the humanoid robot.

**Figure 4:**
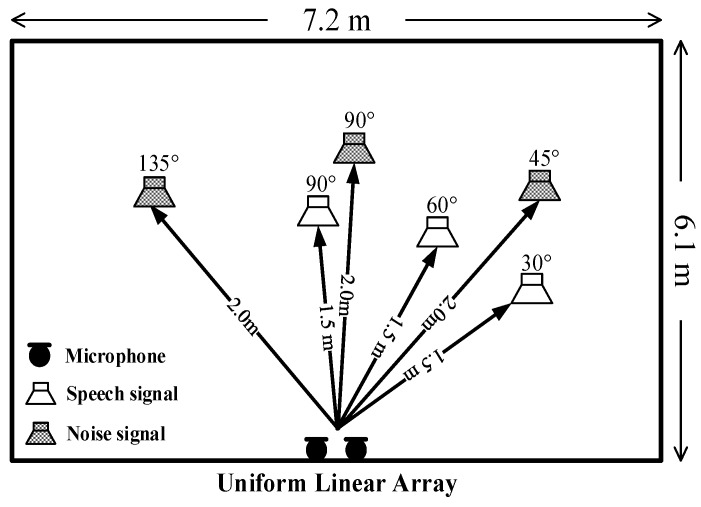
Layout of chamber to the developed system.

Figure 4 shows the layout of the test environment; the length and the width of the experimental chamber are 7.2 m and 6.1 m, respectively. The linear array collects the test speech signal with a sampling rate of 8 kHz. The distance from the robot to the speaker is 1.5 m, and the distance from the robot to the source of the noise is 2 m. The SNR threshold value *ε* is set to 10. In the experiments, three test directions (30°, 60°, and 90°) are utilized to collect the speech signal, and three directions (45°, 90°, and 135°) are used to record the noise signal.

The proposed system utilizes the noisex-92 database [23] to provide the test background noises. The noises in the database are of five types, which are babble noise, car noise, factory noise, pink noise, and white noise. During speech recording, each of the four speakers (three males and one female) utters 30 sentences, each taking approximately three to five seconds. For each type of noise, a total of 1080 sentences are examined with the speakers and the source of noise at varied directions. The linear array firstly records the environmental noise to obtain the initial power value of the noise signal in the test environment. The power value of the noise signal is estimated and updated during the subsequent speech recording.

### 4.2. Evaluation Results

In test speech recording, the system records noisy speech with SNR values of 0 dB, 5 dB, and 10 dB. To compare the quality of enhanced speech with noisy speech, two objective speech quality measures, SNR and segmental SNR, are estimated from the experimental results. Equations (30) and (31) represent the SNR and segmental SNR, where *y*(*t*), *y*′(*t*), *N*, *M*, and *m* are, respectively, the noisy speech, the enhanced speech, the length of the speech signal, the number of frames, and the frame index.

(30) $$SNR=10\log_{10}\frac{\sum_{t=0}^{N-1}y^{2}(t)}{\sum_{t=0}^{N-1}{\left[y(t)-y^{\prime }(t)\right]}^{2}},$$

(31) $$SegSNR=\frac{1}{M}\sum_{m=1}^{M}SNR_{m}$$

Table 1, Table 2 and Table 3 compare the average SNR and segmental SNR values of the noisy speech and enhanced speech using the proposed method. Speech with three SNR values (0 dB, 5 dB, and 10 dB) and five types of noise are used in the experiments. The average SNR values of the enhanced speech exceed the noisy speech by approximately 20 dB to 25 dB. The segmental SNR values of the enhanced speech are also superior to the noisy speech. Both experimental results reveal that the proposed system improves the quality of speech in varied noisy environments.

**Figure 5:**
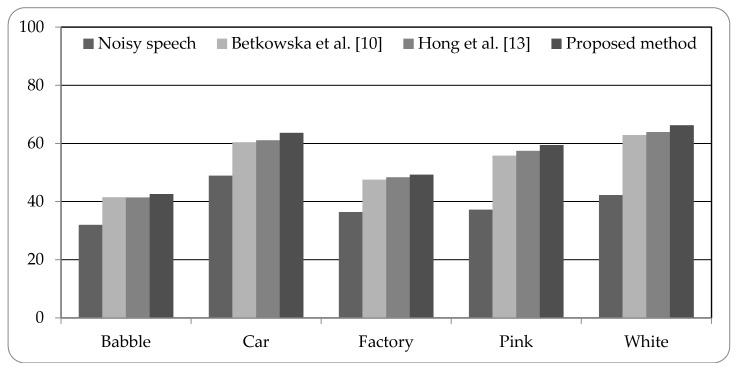
Speech recognition rates of noisy speech (0 dB), related works, and proposed method.

**Figure 6:**
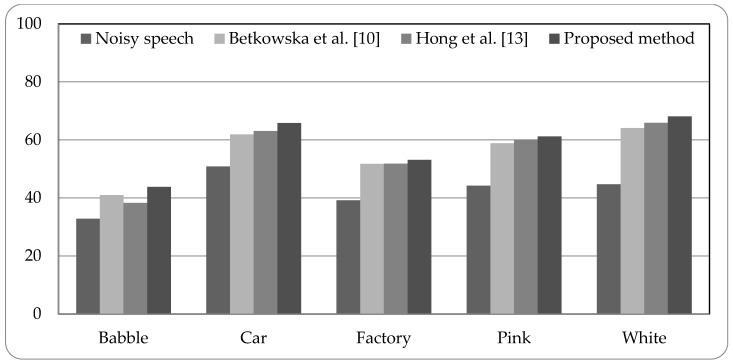
Speech recognition rates of noisy speech (5 dB), related works, and proposed method.

**Figure 7:**
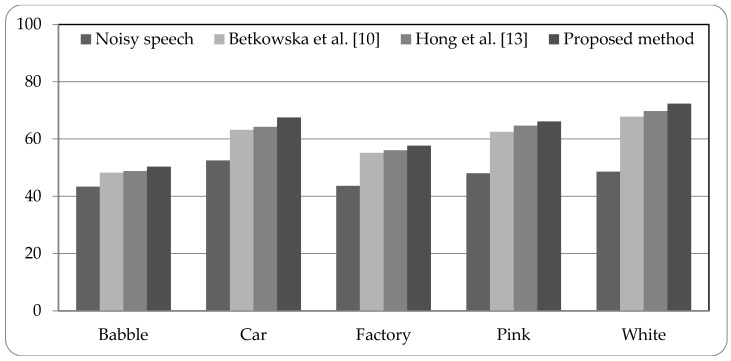
Speech recognition rates of noisy speech (10 dB), related works, and proposed method.

Figure 5, Figure 6 and Figure 7 present the speech recognition rates of the noisy speech, the related works [10,13], and the proposed method. Two related methods based on HMM system are compared with the proposed HMM-based system. Three SNR values of noisy speech with 0 dB, 5 dB, and 10 dB, are examined in experiments. The results indicate that the proposed method can increase the recognition rates than noisy speech by about 15% to 25%. Compared with the related works, the proposed method is superior to the related works; the recognition rates can be better than the related works by 0.94% to 5.52%. The experimental results demonstrate that the proposed system using combined noise separation and speech enhancement methods can effectively remove numerous types of noise and improve the speech quality for speech recognition process.

## 5. Conclusions

This work develops a speech recognition system that can be embedded in a device of interactive robot to recognize the content of speech in noisy environments. The system can be divided into two procedures; the first one is the proposed preprocessing called threshold-based noise detection, and the second one is combined noise reduction. The proposed preprocessing scheme can evaluate the magnitude of noise, to prevent the situation of over-filtering speech when the background noise is slight. In noise reduction, two methods called ICA and SSE are combined to eliminate the effect of noises on the speech signal. ICA is used to separate the noise from the noise-contaminated speech, and the SSE method improves the quality of speech by filtering out the residual noise.

Experimental results indicate that the proposed system can remove the ambient noise and increase the speech recognition rate. The proposed method yields higher SNR value and speech recognition rate than noisy speech. The speech recognition rate is also superior to the related works in experiments. In future work, the system can be combined with several research fields such as acoustic processing, technique of sound source localization, design of home-care service robot, and multimedia analysis [24,25,26], to provide more user-friendly services in application of human-robot interactions.

## Figures and Tables

**Table 1:** Average values of SNR and segmental SNR of noisy speech (0 dB) and enhanced speech.

Noise Type	Noisy Speech	Average SNR of Enhanced Speech	Segmental SNR of Enhanced Speech
Babble	0 dB	24.12 dB	32.67 dB
Car	0 dB	33.31 dB	37.11 dB
Factory	0 dB	26.91 dB	35.15 dB
Pink	0 dB	27.59 dB	35.61 dB
White	0 dB	29.36 dB	35.22 dB

**Table 2:** Average values of SNR and segmental SNR of noisy speech (5 dB) and enhanced speech.

Noise Type	Noisy Speech	Average SNR of Enhanced Speech	Segmental SNR of Enhanced Speech
Babble	5 dB	25.41 dB	32.95 dB
Car	5 dB	37.38 dB	37.21 dB
Factory	5 dB	27.37 dB	35.33 dB
Pink	5 dB	30.83 dB	35.88 dB
White	5 dB	31.38 dB	35.42 dB

**Table 3:** Average values of SNR and segmental SNR of noisy speech (10 dB) and enhanced speech.

Noise Type	Noisy Speech	Average SNR of Enhanced Speech	Segmental SNR of Enhanced Speech
Babble	10 dB	27.67 dB	35.36 dB
Car	10 dB	42.62 dB	39.04 dB
Factory	10 dB	30.78 dB	37.85 dB
Pink	10 dB	36.41 dB	38.88 dB
White	10 dB	35.29 dB	38.14 dB
